# *Longizonitis*, a new nemognathine genus from the Himalayas (Coleoptera, Meloidae)

**DOI:** 10.3897/zookeys.765.24395

**Published:** 2018-06-06

**Authors:** Zhao Pan, Guodong Ren, Marco A. Bologna

**Affiliations:** 1 The Key Laboratory of Zoological Systematics and Application, College of Life Sciences, Hebei University, 071002, Baoding, Hebei Province, China; 2 Dipartimento di Scienze, Università degli studi Roma Tre, Viale G. Marconi 446, 00146, Roma, Italy

**Keywords:** Blister beetles, China, India, new genus, taxonomy

## Abstract

The new blister beetle genus *Longizonitis* Pan and Bologna is described. The genus is referred to the tribe Nemognathini, subfamily Nemognathinae, and its relationships are briefly discussed. It is distributed in southern China (Yunnan, SE Xizang, and probably Fujian) and India (Uttarakhand), in a transitional area between the Palaearctic and Oriental regions. The type species, *Longizonitis semirubra* (Pic, 1911), **comb. n.**, is re-described and illustrated.

## Introduction

The tribe Nemognathini Laporte de Castelnau, 1840, with approximately 530 described species, belonging to 28 genera, is the second most speciose tribe of Meloidae Gyllenhal, 1810 behind the Mylabrini Rafinesque, 1815 has a cosmopolitan distribution (Pinto and Bologna 1999, Bologna and Pinto 2002, Bologna et al. 2013). No comprehensive taxonomic revision or phylogenetic studies have been published on this tribe, but its monophyly was supported in recent papers (Bologna and Pinto 2001, Bologna et al. 2008, Bologna et al. 2013). The taxonomic validity of some genera was debated and possible new genera were highlighted by Bologna and Pinto (2002) and Bologna et al. (2013). In particular, the genus *Zonitis* Fabricius, 1775 seems to have been used as ‘dumping ground’ especially for Afrotropical, Oriental and Neotropical species (Bologna and Pinto 2002, Bologna et al. 2013, Bologna unpublished). Species of this genus have been reviewed for the Nearctic (Enns 1956) and partially for the western Palaearctic (Escherich 1897) regions, while most of the Afrotropical species referred to this genus by Kaszab (1954) actually belong to other genera such as *Palaestra* Laporte de Castelnau, 1840, *Zoltanzonitis* Bologna & Pinto (in press), *Zonitodema* Péringuey, 1909 and *Zonitoschema* Péringuey, 1909 (Bologna unpublished). The Australasian species belong to several genera (see Bologna et al. 2013).

**Figures 1–12.**
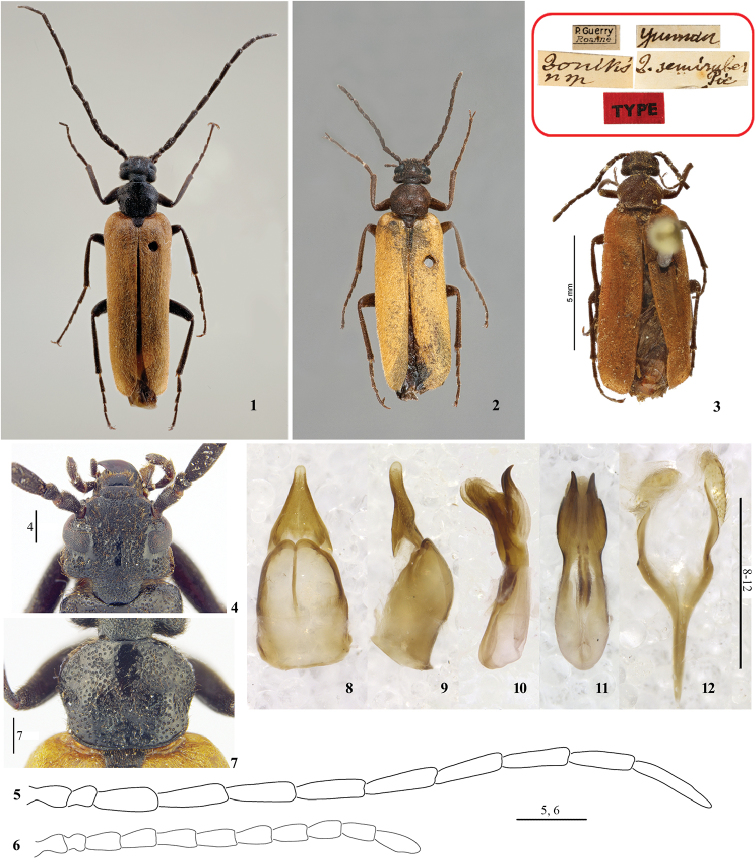
*Longizonitis semirubra* (Pic, 1911), adult **1** habitus, male, Yadong, Xizang (HBUM) **2** habitus, female, Yadong of Xizang (HBUM) **3** holotype and labels, female, Yunnan (MNHN, photographed by Dr Antoine Mantilleri) **4** head, dorsal view, male **5** antenna, male **6** antenna, female **7** pronotum, dorsal view, male **8–12** male genitalia **8** gonoforceps, ventral view **9** gonoforceps, lateral view **10** aedeagus, lateral view **11** aedeagus, ventral view **12** *spiculum gastrale*. Scale bars: 0.5 mm (**4, 7**); 1 mm (**5, 6, 8–12**).

During the study of type specimens of Meloidae housed at MNHN, it was discovered that *Zonitis semirubra* Pic, 1911 (Figs 1–3) does not belong to the genus *Zonitis*. Four other specimens were found at BMNH and HBUM (see below for the abbreviations) and, after the examination of male genitalia, it is clear that *Zonitis semirubra* belongs to a new genus, which is described here together with a re-description of the type species.

## Materials and methods

The following abbreviations used in the text refer to the examined collections:

**BMNH** Natural History Museum, London, United Kingdom;

**HBUM** Hebei University Museum, Baoding, China;

**MNHN** Muséum National d’Histoire Naturelle, Paris, France.

Figures of antennal morphological details were drawn by hand, using a Nikon SMZ1500 stereomicroscope equipped with a camera lucida. Photographs of other morphological details were taken using a Leica M205A stereomicroscope equipped with a Leica DFC450 camera which was controlled using the Leica application suite 4.3. Habitus were taken with a Canon EOS 5D Mark III camera connected to a Canon MP-E 65 mm macro lens.

## Taxonomy

### Longizonitis

Taxon classificationAnimaliaColeopteraMeloidae

Pan & Bologna gen. n.

http://zoobank.org/5781AD01-3736-48F5-BA90-BA2E4AE657E5

#### Type species.

*Zonitis semirubra* Pic, 1911 (originally described as *Zonitis semiruber*) by present designation.

#### Etymology.

From the Latin adjective ‘*longus*’ and *Zonitis*. The name refers to the slender shape of body, which differs from that of several other nemognathine genera.

#### Diagnosis.

*Longizonitis* is clearly distinguishable from other nemognathine genera by the following characters: body elongate, length-width ratio distinctly more than 3.5; antennomere II distinctly shorter than III; elytra not reduced in size, only slightly dehiscent apically; tarsal claws with ventral blade narrow, its greatest width slightly more than half the basal width of dorsal blade; female with two metatibial spurs similar in shape and size, male external metatibial spur as in female, inner one stick-liked and only half width of external one; male ventrite VI completely divided, that of female V-emarginate; aedeagus without dorsal hooks, but with two sclerotised ventral lobes, curved posteriad; gonostyli almost completely fused, gonocoxal plate longer than gonostyli.

#### Description.

Head short, subrectangular, head width at temples slightly greater than at eyes, frons not depressed, surface with dense, large and shallow punctures (Fig. 4). Eyes normal in size, only extending ventrally to outer margin of maxillae on underside of head, slightly emarginate on fore margin near base of antenna. Mandibles robust and long, extending beyond fore margin of labrum; galeae short and fringed (as in fig. 71, Bologna and Pinto 2002); maxillary palpi four segmented, palpomeres not elongate, last palpomere not widening at apex. Antennae with eleven antennomeres, filiform, elongate and slender; antennae slightly longer than elytral length in male (Figs 1, 5), and shorter in female (Figs 2, 3); male antennomere II short, subglobose, about as wide as long, apical antennomeres equal in width to basal ones; female antennomeres distinctly shorter than in male, XI almost suboval (Figs 2, 3, 6).

Pronotum wider than long, punctures as on head, slightly more scattered (Fig. 7). Elytra elongate, normal, not reduced in size, slightly dehiscent apically on inner margin; each elytron with four weak costae and with dense short setae. Hind-wings present and regularly developed. Legs not modified in both sexes; both female metatibial spurs wide, spatulate and concave dorsally, similar and subequal in length and width; male external metatibial spur as in female, inner spur stick-liked and only half the width of external spur; tarsal claws with ventral blade narrow, its greatest width slightly more than half basal width of dorsal blade (as in fig. 100, Bologna and Pinto 2002); dorsal blade of claw with two rows of teeth along its ventral margin, outer row incomplete.

Male ventrite VI deeply cleft to base and completely divided longitudinally (as in fig. 105, Bologna and Pinto 2002); slightly V-emarginate in female. Male gonostyli almost completely fused, slightly separate at apex; gonocoxal plate longer than wide and longer than gonostyli, gibbous ventrally (Figs 8, 9). Aedeagus subcylindrical, without dorsal hooks, but with two sclerotised ventral lobes curved posteriorly; endophallus without hook (Figs 10, 11).

#### Distribution.

Southern China, northwestern India.

#### Relationships.

The new genus differs from all known Nemognathinae taxa and shows mixed distribution of character states; for this reason, their relationships remain difficult to define. It clearly belongs to the tribe Nemognathini and not to the Palaestrini Bologna, Turco & Pinto, 2013 or Horiini Latreille, 1802, due to the cylindrical shape of the aedeagus and unmodified mandibles (see Bologna et al. 2013). Among the tribe Nemognathini, the antennomere II distinctly shorter than III (typical of Palaestrini) is an uncommon condition, present only in a few taxa, though notably occurring in the Afrotropical genus *Zoltanzonitis* (Bologna and Pinto, in press). The shortened antennomere II is also present in the *Nemognatha*-lineage as defined by Bologna et al. (2103) (*Palaestrida* White, 1846, some Nearctic *Nemognatha* Illiger, 1807), in which, however, lack ventral sclerotized lobes of aedeagus.

Ventral sclerotised lobes are present in all New World *Zonitis*, *Pseudozonitis* Dillon, 1952 and *Gnathium* Kirby, 1818 species, and some Palaearctic species of *Zonitis*; however, the short antennomere II is never represented in the American species.

In the new genus, galeae are neither penicillate nor greatly modified, a plesiomorphic condition more similar to that of Nemognathini of the sitarine lineage than that of typical lineage (see Bologna et al. 2008 for the lineages definition). Additionally, the shape of pronotum differs from that of most Nemognathini, except for some of the sitarine lineage.

### Longizonitis semirubra

Taxon classificationAnimaliaColeopteraMeloidae

(Pic, 1911) comb. n.

Zonitis semiruber Pic, 1911: 101 (type locality: Yunnan, China; type depository: MNHN); Borchmann, 1917: 164; 1941: 23.
Zonitis semirubra : Hua, 2002: 131.
Zonitis (Zonitis) semirubra : Bologna, 2008: 411.

#### Diagnosis.

This species, the only known member of the genus *Longizonitis*, can be diagnosed by the generic diagnosis given above. Because Pic’s (1911) description is very short and provides limited morphological data, a detailed re-description of the species based on new specimens from Xizang, Yadong (China) and Uttarakhand, Kumaon (India), is provided below.

#### Re-description.

Characters of the genus (see above) with the following details. Body (Figs 1–3) without metallic reflections, black except elytra reddish brown and last three or all abdominal ventrites yellow. Body with dense, yellow-brown, short setae. Body length (apex of mandibles – apex of elytra): 9.4–12.0 mm; body width (elytral width at widest point): 2.7–3.1 mm.

Head slightly wider than long (from fronto-clypeal suture to posterior margin of head), with maximum width at level of temples (Fig. 4). Punctures separated by less than their width, with a longitudinal impunctate area on centre of frons and around eyes (Fig. 4). Temple curved posteriorly and almost as long as length of eye. Clypeus slightly narrower than interocular width, rounded on sides, posteriorly with punctures similar to those of frons and anteriorly almost smooth and slightly sloping; labrum scarcely narrower than clypeus, rounded on sides, fore margin almost straight in both sexes, medially not depressed. Mandibles curved and progressively narrowed on apical half. Antennomere I longer than II, III–X subcylindrical and slender, similar in length, XI nearly 1.5 times as long as X, subcylindrical but narrowed in apical third; male antennomeres I and II as long as in female, III–XI considerably longer than in female (Figs 5, 6).

Pronotum slightly wider than head at temples, widest just in front of middle, rounded on sides, distinctly narrowed anteriorly and slightly posteriorly; two bulging areas behind the widest point (Fig. 7). Elytra unicolourous, elongate, approximately 2.8 times as long as wide. Legs slender; protibiae and mesotibiae with two spurs in both sexes, both slender, pointed apically and similar in length; metatibial spurs both spatulate in female, male external metatibial spur as similar as female, inner one stick-like and only half width of external one; tarsomeres in both sexes without pads; protarsi evidently longer than protibiae, protarsomeres longer than wide; teeth on ventral margin of dorsal blade of tarsal claws present only in basal half on outer row.

Male gonoforceps (Fig. 8) in ventral view subtriangular; *spiculum gastrale* Y-shaped (Fig. 12).

#### Material examined.

***Type.*** Holotype female, “P. Guerry // *Roanne*” (white, rectangular, printed), “Yunnan” (white, rectangular, handwritten), “*Zonitis* n. sp.” (white, rectangular, handwritten), “*Z. semiruber* Pic” (white, rectangular, handwritten), “TYPE” (red, rectangular, printed, added subsequently) (MNHN; as Fig. 3).

***Other specimens.*** 1 male, 2 females, “2005-7-22 // 西藏亚东 // 石爱民 // 河北大学博物馆 [2005-7-22 // China, Xizang, Yadong // Shi Aimin // Hebei University Museum]” (white, rectangular, printed) (HBUM; as Figs 1–2). 1 male, 1 female, “2017-VI-23 // 西藏亚东下亚东 // 邱见玥、许浩 // 河北大学博物馆 [2017-VI-23 // China, Xizang, Yadong, Xiayadong // Qiu Jianyue & Xu Hao leg. // Hebei University Museum]” (white, rectangular, printed) (HBUM). 1 male, “Kumaon Ramgahr, 6000’ [= 1828 m ca.], 21–26.viii.(19)18, Fletcher coll:, ex coll. Pasa Inst., B.M. 1924-220” (BMNH).

#### Distribution.

**Figure 13.**
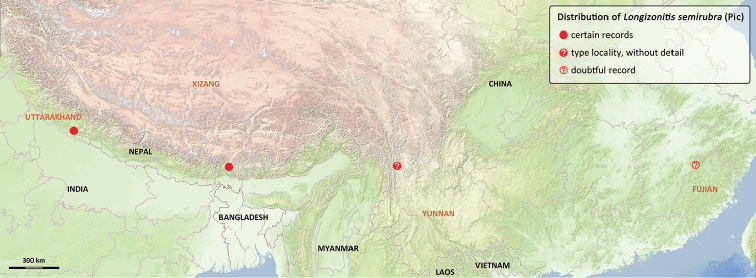
Distribution of *Longizonitis semirubra* (Pic).

(Fig. 13) CHINA: Fujian? (Borchmann 1941, Hua 2002), Yunnan (Pic 1911, Borchmann 1917, Hua 2002; MNHN), SE Xizang (HBUM). INDIA: Uttarakhand (BMNH).

#### Remarks.

The holotype and one of the female specimens from Yadong have a dark reddish body colouration that could be caused by the following reasons: 1) the curation and conservation conditions would make the colour change from black to dark reddish over time; 2) these individuals may still be somewhat teneral, and the integument was not completely sclerotized and/or pigmented.

The name of this species was emended as *semirubra* by Hua (2002) because *Zonitis* is a female genus and in Latin the adjective *ruber* is masculine and *rubra* is feminine.

Borchmann (1941) recorded three specimens from “Kwangtsch-Fukien”, which corresponds to Hangchuan in the Fujian province of China. However, this locality is far from others Chinese recorded (Yunnan, Xizang) and it is doubtful because it is located in tropical coastal region of China, while other localities are in high mountain regions. We are not sure if this record is correct because Borchmann (1941) indicated that the identification was in doubt due to the short description published by Pic (1911). This specimen is probably housed at the Bonn Museum (Germany).

## Supplementary Material

XML Treatment for Longizonitis

XML Treatment for Longizonitis semirubra
